# FCGR3A V158F gene polymorphism and trastuzumab response in HER2-positive breast cancer patients

**DOI:** 10.1038/s41598-024-76024-6

**Published:** 2024-10-29

**Authors:** Marwa A. Abdel-Wahed, Ghada Sadek Sabbour, Amira I. Hamed, Mohammed Sabry EL Kady, Shaimaa Khalil Mohammed, Menat Allah Ali Mahmoud Shaaban

**Affiliations:** 1 Department of Clinical Pathology, Faculty of Medicine, Ain Shams University, 38 Abbassyia Square, Cairo, Egypt; 2 Department of Oncology, Faculty of Medicine, Ain Shams University, Cairo, Egypt

**Keywords:** *FCGR3A*, Gene polymorphism, Trastuzumab, Cancer, Diseases, Oncology

## Abstract

Breast cancer is considered a multifactorial disease, with genetic factors playing an important role in diagnosis and treatment. *FCGR3A* encodes the receptor for the Fc portion of immunoglobulin G that has been linked to the trastuzumab response. Our study aimed to investigate the association of *FCGR3A-V158F* gene polymorphism with breast cancer and to evaluate the impact of *FCGR3A-V158F* gene polymorphism on trastuzumab response in HER2-positive breast cancer patients. The study was conducted on eighty breast cancer patients who were collected from the Department of Oncology at Ain Shams University Hospitals; in addition, twenty age-matched healthy subjects were taken as a healthy control group. Patients were further sub-classified according to their responses. The study showed that there were no statistically significant differences between patients and controls regarding *FCGR3A-V158F* gene polymorphism genotypes. However, there was a significant association between the concordance of this polymorphism and the response to trastuzumab therapy among the patient’s group. V/V is associated with better treatment response and overall survival (OS) compared to F/V and F/F alleles. Assessment of FCGR3A-V158F gene polymorphism might be useful in making a treatment decision in HER2-positive breast cancer patients.

## Background

Breast cancer is the most common cancer, accounting for 2.26 million incident cases in 2020, and a leading cause of death in women worldwide^1^. In Egypt, it constitutes 33% of female cancer cases, and more than 26,845 new cases are diagnosed each year^2,3^. Breast cancer is estimated to be the second-leading cause of death from cancer in Egyptian women^2^.

Breast cancer is considered a multifactorial disease due to the combined effect of genetic and environmental factors, with genetic factors playing an important role in diagnosis and the course of the disease^4^. About 20–25% of breast cancer patients are human epidermal growth factor receptor 2 (HER2+; ErbB2/neu) positive, and its overexpression is associated with a worse prognosis in breast cancer^4,5^.

Clinical trials have demonstrated that trastuzumab can significantly increase disease-free survival, and overall survival, and improve the prognosis of HER2-positive breast cancer patients^6,7^. However, the response rate of trastuzumab varies between patients^7^. The RECIST is a standardized criterion in solid tumors and become globally accepted for evaluating the tumor response^8,9^.

The main trastuzumab mechanism for attacking the tumor cells is to trigger the host immune system through a receptor the Fc portion of immunoglobulin G, which is encoded by the *FCGR3A* gene, and it is involved in the removal of antigen-antibody complexes from the circulation, as well as other responses, including antibody-dependent cellular cytotoxicity (ADCC)^10^.

The presence of polymorphisms in the *FCGR3A* gene results in variants with a different signaling function that bind to the Fc portion of immunoglobulin G with a varying affinity affecting the ADCC potency^11^, These variations have an impact on the success of immunotherapy among cancer patients. The *FCGR3A-V158F* polymorphisms are hypothesized to potentially affect the trastuzumab therapy^12^.

The *FCGR3A-V158F* (rs396991) is a single nucleotide substitution (SNP) from G to T at DNA nucleotide position 559 of the *FCGR3A* gene, resulting in a substitution of valine (V) to phenylalanine (F) at amino acid position 158 in the immunoglobulin G -binding domain^13,14^. The presence of the F/F genotypes has been linked to higher response rates and progression-free survival^12,15^.

The frequency of these polymorphisms varies by race in the general population, including cancer patients and healthy individuals^11^. Though it hasn’t been proven or verified, it has been assumed that African patients may not respond to trastuzumab treatment in comparison to non-African patients as it has been reported that higher *FCGR3A-V158F* frequencies among Africans than Caucasians^12,16^. Although polymorphisms, such as *FCGR3A-V158F*, have been extensively studied^14^, an overview of *FCGR3A-V158F* polymorphisms within the Egyptian population is lacking and the association of this polymorphism with trastuzumab benefit for patients with breast cancer is not clear. Accordingly, this study aims investigate the association between *FCGR3A-V158F* gene polymorphism and response to trastuzumab in HER2-positive breast cancer patients.

## Methods

### Patients and sample acquisition

The current study was approved by the Institutional Ethics Committee (Ethical Committee’s reference number: FMASU 357/2019) (Cairo, Egypt). Written informed consent was collected from all involved participants. The sample size was calculated using Epi Info and according to *Gavin et al.*^15^, assuming a sample proportion of 50% (to give maximum size) and with 95% confidence interval and a confidence interval width of 10%, eighty individuals are needed.

Peripheral venous blood samples were retrieved from eighty consecutive patients who were recently diagnosed with breast cancer and histologically confirmed to have HER2 positive breast cancer (3+) before initiation of any therapy, at the Ain Shams University Hospitals (Cairo, Egypt). In addition, twenty control blood samples were obtained from apparently healthy females attending the clinic for routine screening. Eligible controls were healthy age-matched females with no prior previous history of breast cancer or any other malignancies or family history of breast cancer. In addition, breast cancer was excluded by clinical breast examination and mammography. Both groups of breast cancer patients and controls developed menarche normally and started their menstrual periods after age 12.

Regarding breast cancer patients, the diagnosis of breast cancer was based on histopathological examination of breast tissue biopsy and immunophenotyping for HER2 receptors by immunohistochemistry. Estrogen receptor (ER)/progesterone receptor (PR) positivity was determined by immunohistochemistry analysis of the number of positively stained nuclei (≥ 10%), and hormone receptor (HR) positivity was defined as being either ER + and/or PR+. Exclusion criteria were patients with any other comorbidity contraindicating chemotherapy and patients having other types of malignant tumors other than breast cancer.

All breast cancer patients received the same preoperative regimen of neoadjuvant chemotherapy and anti-HER2 according to the following NCCN regimen; doxorubicin (60 mg/m^2^ IV) and cyclophosphamide (600 mg/m^2^ IV) for four cycles, followed by paclitaxel (175 mg/m^2^ IV), with the addition of trastuzumab (6 mg/kg) every 21 days for four cycle then they underwent surgery. After that patients were given a complete one year of trastuzumab therapy^17^. Assessment of trastuzumab response was performed by clinical, physical examination, and mammography for all the patients at the same time preoperative after completion of the four cycles of trastuzumab therapy. The response evaluation criteria in solid tumors (RECIST) 1.1 was used for disease response assessment. Patients who showed a complete response or partial remission were considered responders, and patients who presented with stable disease or progressive disease were regarded as non-responders^18^.

Three milliliters (3 mL) of venous blood were collected, on enrollment from all subjects under complete aseptic conditions, into a sterile K3 EDTA vacutainer and stored at -70 °C to be used for the detection of *FCGR3A-V158F* gene polymorphism. Repeated freezing and thawing were avoided.

### Genomic DNA purification

Genomic DNA was extracted using the Gene JET whole blood genomic DNA purification mini kit (ThermoFisher Scientifc Inc, Bleiswijk, Netherlands), according to the manufacturer’s instructions.

### FCGR3A-V158F (rs396991) genotyping

The extracted DNA was amplified using TaqMan universal master mix and ready-made TaqMan SNP Genotyping assay (assay ID C_25815666_10; ThermoFisher Scientifc Inc, Bleiswijk, Netherlands) (https://www.thermofisher.com/order/genome-database/details/genotyping/C__25815666_10#assay-details-section). TaqMan SNP genotyping assay was diluted from 40X to a 20X working stock with 1X TE buffer. The mixture was vortexed, and then centrifuged. The components of the reaction mix in each PCR Reaction were as the followings: 10µL TaqMan universal master mix, 0.5 µL 20x working stock of TaqMan SNP Genotyping assay, 7.5 µL DNase-free water, and 2µL genomic extracted DNA 0.2 µmol/L. Thermal cycling was performed in a DTlite real-time PCR system (DNA technology, Russia) under the following conditions: Initial denaturation at 95 °C for 10 min followed by 50 cycles of 95 °C for 15 s, 60 °C for 60 s, and finally 60 °C for 7 min.

### Conventional PCR and Sanger sequencing

Three blood samples of breast cancer patients were amplified by conventional PCR using primer pairs for the region of *FCGR3A-V158F* using forward: 5’- ACAGAGCTGCAAACCTACCC − 3’ and reverse: 5’- CAGCTCTTCCCCAAAGGGAG − 3’. Thermal cycling was performed in a thermocycler (3Prime thermal cycler; Techne, Cole-Parmer, United Kingdom) under the following conditions: Initial denaturation at 95 °C for 4 min followed by 35 cycles of 95 °C for 30 s, 58 °C for 30 s, and 72 °C for 60 s, then finally 72 °C for 10 min. The PCR products were purified using a QIAquick gel extraction kit (Qiagen) and then sequenced on ABI 310-Analyser (Applied Biosystems; Thermo Fisher Scientific, Inc.). BigDye^®^ Terminator v3.1 cycle sequencing was used to perform the sequencing reaction according to the manufacturer’s protocol.

### Statistical analysis

Statistical analysis was performed using the SPSS software version (V. 26.0, IBM Corp., USA, 2019). The values were expressed as median and IQR for the non-parametric data, while categorical variables were summarized using frequency measures. Kruskall Wallis test and chi-square test were used for comparative analysis.

Testing for Hardy-Weinberg Equilibrium (HWE) estimates the independent segregation of alleles in a population. Allo-genotype frequencies were calculated by the gene-counting method and were checked for Hardy–Weinberg analysis through the chi-square test (X2 test), *p* < 0.05 was considered significant. The multivariate logistic regression analysis was used to assess the independent factors including the different demographic and clinicopathological features such as age, menopausal status, family history of breast cancer, site of the tumor, and the status of ER and PR.

Survival analysis was only performed using data from trastuzumab-treated samples, using both progression-free survival (PFS) (in months) and overall survival (OS). Survival curves are based on Kaplan- Meier estimates, and the log-rank *p*-value is shown for the difference in survival.

## Results

Our study included 80 confirmed newly diagnosed breast cancer cases with a median age of 52 (IQR, 45-55.75) years, compared with 20 apparently healthy control subjects with a median age of 56 (IQR, 49–57) years. The *FCGR3A-V158F* genotype was determined in all subjects and the genotyping frequencies were in compliance with Hardy-Weinberg equilibrium (*p* = 0.07).

Regarding the contraceptive characteristics of the enrolled control subjects, 12 (60%) did not use any contraceptive methods, 3 (15%) used oral contraceptive pills (OCP), and 5 (25%) used intrauterine devices (IUD). These data were compared to the enrolled patients, 49 (61.2%) did not use any contraceptive methods, 17 (17.5%) used OCP, and 17 (21.3%) used IUD (*p* > 0.05). Regarding the menopausal status, 11 (55%) control subjects were post-menopausal and 45 (56.3%) patients were post-menopausal. No statistically significant differences were observed between the two groups regarding the studied demographic characteristics (*p* > 0.05).

The clinicopathologic characteristics of the enrolled patients are summarized in Table 1. No significant association was found between the genotype frequencies of *FCGR3A-V158F* and patients’ age, menopausal status, family history of breast cancer, use of contraception, tumor size, the TNM staging, site of the tumor, mammograph, lymph nodes, and histopathological grade, as well as the different status of ER and PR (*p* > 0.05), as in Table 2. The median OS was 14 vs. 16 vs. 22 months for the patients with genotyping F/F vs. F/V vs. V/V, respectively (*p* = 0.005). Regarding the PFS, the median PFS was 6 vs. 10 vs. 22 months for the patients with genotyping F/F vs. F/V vs. V/V, respectively (*p* = 0.001) (Table 2).

**Table 1:** Clinicopathological characteristics of breast cancer patients.

Parameter		Breast cancer patients (*n* = 80)
		n (%)
Tumor size (cm)	**< 5**	55 (68.75%)
	**> 5**	25 (31.25%)
TNM staging	II A	22 (27.5%)
	II B	23 (28.7%)
	III A	35 (43.8%)
Site of the tumor	Right	60 (75.0%)
	Left	20 (25.0%)
Mammography	Left breast mass BIRADS 3	2 (2.5%)
	Left breast mass BIRADS 4	8 (10.0%)
	Left breast mass BIRADS 5	10 (12.5%)
	Right breast mass BIRADS 4	39 (48.8%)
	Right breast mass BIRADS 5	21 (26.2%)
Lymph nodes	Negative	22 (27.5%)
	Positive	58 (72.5%)
Histopathology	Invasive ductal carcinoma Grade 2	64 (80.0%)
	Invasive ductal carcinoma Grade 3	16 (20%)
ER	Negative	38 (47.5%)
	Positive	42 (52.5%)
PR	Negative	45 (56.2%)
	Positive	35 (43.8%)
HER2	Negative	0 (0.0%)
	Positive	80 (100.0%)
Trastuzumab Response	Complete response	31 (38.75%)
	Partial response	13 (16.25%)
	Progressive disease	16 (20.0%)
	Stable disease	20 (25.0%)

*ER* estrogen receptors; *HER2* human epidermal growth factor receptor 2; *PR* progesterone receptors; *TNM* tumor, nodes, and metastasis.

**Table 2:** *FCGR3A-V158F* genotyping characteristics among breast cancer patients.

Parameter		F/F (*n* = 15)	F/V (*n* = 49)	V/V (*n* = 16)	*P*-value
		*n*(%)/ median (IQR)	*n*(%)/ median (IQR	*n*(%)/ median (IQR	
Age (years)		52 (45–56)	52 (45.5–55.5)	49.5 (35.3–56.5)	0.598
Pre-menopausal		7 (46.7%)	20 (40.8%)	8 (50.0%)	0.788
Post-menopausal		8 (53.3%)	29 (59.2%)	8 (50.0%)	
Family history	Negative	12 (80.0%)	31 (63.3%)	9 (56.2%)	0.352
	Positive	3 (20.0%)	18 (36.7%)	7 (43.8%)	
Contraception	No	6 (40.0%)	33 (67.3%)	10 (62.5%)	0.333
	OCP	5 (33.3%)	7 (14.3%)	2 (12.5%)	
	IUD	4 (26.7%)	9 (18.4%)	4 (25.0%)	
Tumor size (cm)	**< 5**	8 (53.3%)	36 (37.5.5%)	11 (68.8%)	0.338
	**> 5**	7 (46.7%)	13 (26.5%)	5 (31.3%)	
TNM staging	II A	7 (46.7%)	9 (18.4%)	6 (37.5%)	0.229
	II B	3 (20.0%)	16 (32.7%)	4 (25.0%)	
	III A	5 (33.3%)	24 (49.0%)	6 (37.5%)	
Site of the tumor	Right	12 (80.0%)	35 (71.4%)	13 (81.2%) 3 (18.8%)	0.867
	Left	3 (20.0%)	14 (28.6%)		
Mammography	Left breast mass BIRADS 3	0 (0.0%)	2 (4.1%)	0 (0.0%)	0.908
	Left breast mass BIRADS 4	2 (13.3%)	5 (10.2%)	1 (6.2%)	
	Left breast mass BIRADS 5	1 (6.7%)	7 (14.3%)	2 (12.5%)	
	Right breast mass BIRADS 4	8 (53.3%)	24 (49.0%)	7 (43.8%)	
	Right breast mass BIRADS 5	4 (26.7%)	11 (22.4%)	6 (37.5%)	
Lymph nodes	Negative	6 (40.0%)	11 (22.4%)	5 (31.3%)	0.383
	Positive	9 (60.0%)	38 (77.6%)	11 (68.7%)	
Histopathology	Invasive ductal carcinoma grade 2	13 (86.7%)	40 (81.6%)	11 (68.7%)	0.316
	Invasive ductal carcinoma grade 3	2 (13.3%)	9 (18.4%)	5 (31.3%)	
ER	Negative	8 (53.3%)	22 (44.9%)	8 (50.0%)	0.828
	Positive	7 (46.7%)	27 (55.1%)	8 (50.0%)	
PR	Negative	12 (80.0%)	23 (46.9%)	10 (62.5%)	0.067
	Positive	3 (20.0%)	26 (53.1%)	6 (37.5%)	
OS (months)		14 (10–20)	16 (10–22)	22 (20–24)	0.005
PFS (months)		6 (6–6)	10 (6–22)	22 (18.5–24)	0.001

*ER* estrogen receptors; *HER2* human epidermal growth factor receptor 2; *IUD* intrauterine devices; *OCP* oral contraceptive pills; *PR* progesterone receptors; *OS* overall survival; *PFS* progression free survival; *TNM* tumor, nodes, and metastasis.

The multivariate logistic regression analysis shows that the demographic and clinicopathologic factors including age, family history, menopausal status, tumor site, and hormonal status of ER and PR had a non-significant impact as confounding factors (*p* = 0.601, *p* = 0.645, *p* = 0.233, *p* = 0.101, *p* = 0.694, *p* = 0.496, *p* = 0.541, respectively).

Comparative statistics of the genotypes and allelic frequencies of the *FCGR3A-V158F* polymorphism between breast cancer patients and healthy controls are presented in Table 3. No statistically significant differences were observed regarding *FCGR3A-V158F* genotypes and allele frequencies between the studied patients and controls (*p* > 0.05). Also, no statistically significant differences were observed between V/V and F/V genotypes or between F/F and F/V genotypes (*p* > 0.05).

**Table 3:** Genotypic and allelic distribution of *FCGR3A-V158F* gene polymorphism in breast cancer patients and healthy controls.

Parameter		Breast cancer patients (*n* = 80)	Healthy controls (*n* = 20)	χ2	*P*-value
		*n* (%)	*n* (%)		
*FCGR3A-V158F* genotypes	**F/F**	15 (18.8%)	5 (25.0%)	0.846	0.655
	**F/V**	49 (61.2%)	10 (50.0%)		
	**V/V**	16 (20.0%)	5 (25.0%)		
*FCGR3A-V158F* alleles distribution	**F** **V**	79 (49.3%) 81(50.6%)	20 (50%) 20 (50%)	0.005	0.943

*F* phenylalanine; *V* valine; *χ2* chi-square test.

We investigated the genotypic association of *FCGR3A-V158F* and the response to trastuzumab therapy as presented in Table 4, the wild type (F/F) was present in 3/44 (6.8%) responders and in 12/36 (33.3%) non-responders. On the other hand, the mutant homozygous genotype (V/V) was detected in 13/44 (29.6%) responders and in 3/36 (8.4%) non-responders. In addition, the mutant heterozygous (F/V) was present in 28/44 (63.6%) responders and in 21/36 (58.3%) non-responders (*p* = 0.003).

**Table 4:** Genotypic and allelic distribution of *FCGR3A-V158F* gene polymorphism among trastuzumab responders and non-responders.

Parameter		Responders ( *n* = 44)	Non-responders (*n* = 36)	χ2	*P*-value
		*n* (%)	*n* (%)		
*FCGR3A-V158F* genotypes	**F/F**	3 (6.8%)	12 (33.3%)	11.97	0.003
	**F/V**	28 (63.6%)	21 (58.3%)		
	**V/V**	13 (29.6%)	3 (8.4%)		
*FCGR3A-V158F* alleles distribution	**F** **V**	34(38.6%) 54 (61.4%)	45 (62.5%) 27 (37.5%)	9.002	0.002

*F* phenylalanine; *V* valine; *χ2* chi-square test.

Also, a statistically significant difference was found between the response grades and FCGR3A-V158F genotypes. 64.5% of patients with complete treatment response (20/31) had F/V genotypes, and 35.5% of patients with complete treatment response (11/31) had V/V genotypes. While 56.3% of patients with progressive disease response (9/16) had F/F genotypes, 37.5% of patients with progressive disease response (6/16) had F/V genotype and 6.3% (one patient only) had V/V genotype (*p* = 0.000) as in Table 5.

**Table 5:** The response grades and the *FCGR3A-V158F* gene polymorphism.

Parameter		Response grade				χ2	*p*-value
		Complete response (*n* = 31)	Partial response (*n* = 13)	Progressive disease (*n* = 16)	Stable disease (*n* = 20)		
*FCGR3A-V158F* polymorphism	**F/F**	0 (0.0%)	3 (23.1%)	9 (56.3%)	3 (15.0%)	26.604	0.000
	**F/V**	20 (64.5%)	8 (61.5%)	6 (37.5%)	15 (75.0%)		
	**V/V**	11 (35.5%)	2 (15.4%)	1 (6.3%)	2 (10.0%)		

*F* phenylalanine; *V* valine; *χ2* chi-square test.

**Fig. 1:**
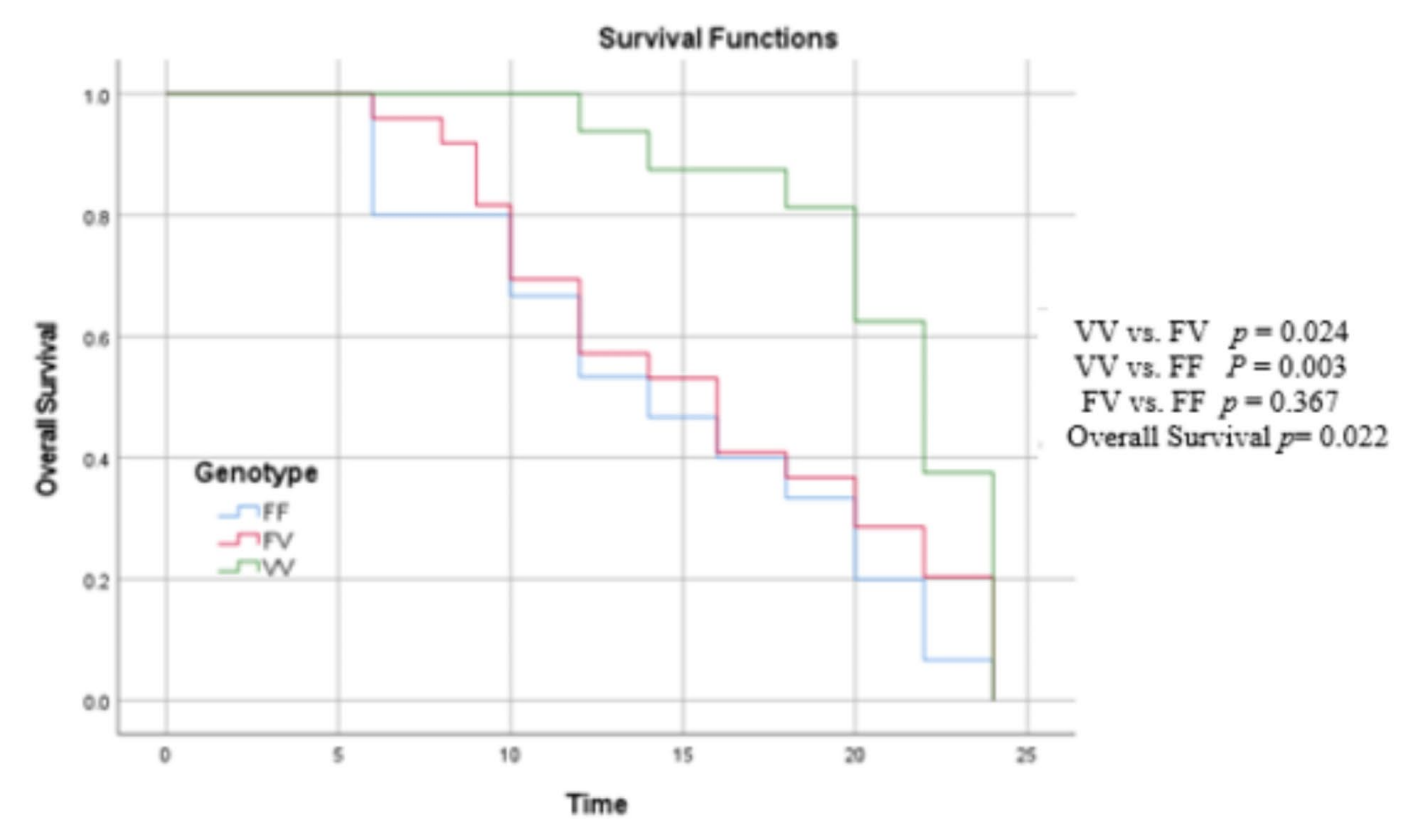
Kaplan Meier curve of OS for *FCGR3A-V158F* genotyping.

Kaplan-Meier analysis of OS (in months) for the *FCGR3A-V158F* genotyping is shown in Table 6; Fig. 1. A significant association was observed between OS and the genotypic variation of *FCGR3A-V158F* (*p* = 0.022). OS in patients with the V/V genotype was significantly higher than in patients with the F/F genotype (*p* = 0.003). Also, OS in patients with the V/V genotype was significantly higher than those with the F/V genotype (*p* = 0.024), while OS in patients with the F/V vs. F/F genotype showed a non-statistically significant difference, *p* = 0.367.

**Table 6:** Kaplan-Meier analysis of the OS (in months) for *FCGR3A-V158F* genotyping.

Parameter		Median OS (months)	95% confidence intervals		Log Rank (Mantel-Cox)	
			Lower	Upper	χ2	*p*-value
*FCGR3A-V158F* polymorphism	**F/F**	14	8.319	19.681	7.622	0.022
	**F/V**	16	12.628	19.372		
	**V/V**	22	20.102	23.898		
	**Overall**	16	12.813	19.187		

*F* phenylalanine; *OS* overall survival; *V* valine; *χ2* chi-square test.

## Discussion

Human epidermal growth factor receptor-2 is a transmembrane receptor implicated in tumor replication, invasion, and dissemination. HER2 overexpression is associated with a worse prognosis in breast cancer patients^19,20^. Trastuzumab combined with chemotherapy became a basic standard treatment for both metastatic and early-stage HER2-positive breast cancer^21^.

To our knowledge, the study of the polymorphism of *FCGR3A* gene in breast cancer and the effect of their allele frequency is still lacking. Previous studies have shown that genetic polymorphism of them *FCGR3A-V158F* rs396991 may affect the response to trastuzumab treatment in HER2-positive breast cancer patients^12,14,22^. However, due to the differences in ethnicity, country, and experimental methods, the results of the studies investigating the relation between *FCGR3A-V158F* rs396991 polymorphism and the response to trastuzumab in patients with breast cancer have been controversial^22^.

A positive association of this genetic polymorphism with clinical outcomes in patients treated with trastuzumab would support the use of genotyping to preselect patients most likely to respond to trastuzumab treatment and further engineering of monoclonal antibodies with increased affinity for the FCGRs. In this study, we evaluated the association between *FCGR3A-V158F* gene polymorphism and response to trastuzumab in HER2-positive breast cancer patients.

We noticed that all the enrolled patients had invasive ductal carcinoma. Also, 58% of our patients had positive lymph nodes; these findings were previously demonstrated by *Abdelaziz et al.* who reported an invasive nature of breast cancer in Egyptian females, which may be linked to patient factors such as lack of knowledge regarding symptoms of the disease and barriers in the health care systems for cancer management such as lack of access to hospitals and high cost of disease screening^3^. In most cases, the time taken from developing symptoms until commencing treatment is more than 6 months, which is enough time for breast cancer to progress and upstage^3,23^.

Our study investigated the relationship between the *FCGR3A-V158F* polymorphism and the clinicopathological characteristics of breast cancer patients, including age, family history, TNM staging, tumor size, lymph nodes, histopathological grade, and hormonal receptor status. The *FCGR3A-V158F* gene genotype did not show any statistically significant variation with the different clinicopathological characteristics of our breast cancer patients. These findings were in agreement with *Norton et al.* who did not find any correlation between *FCGR3A-V158F* polymorphism and clinicopathological parameters of the disease^24^.

To our knowledge, this is the first study investigating the genotypic and allelic frequency of the studied *FCGR3A-V158F* rs396991 polymorphism among breast cancer patients in comparison to healthy controls. Data of the present study revealed that the frequencies of the *FCGR3A-V158F* wild genotype (F/F), homozygous genotypes (V/V), as well as (F) allele, were higher in healthy controls when compared to breast cancer patients (F/F: 25% vs. 18.8%; V/V: 25% vs. 20%; and F allele: 50% vs. 49.3%). On the other hand, frequencies of heterozygous genotypes (F/V), and (V) alleles were higher among breast cancer patients compared to healthy controls (F/V: 61.2% vs. 50% and V allele: 50.6% vs. 50%). However, this difference in the genetic frequency between the two groups did not reach a statistically significant difference.

Identification of involved gene polymorphism related to response to trastuzumab is essential for treatment personalization. So far, no previous study has reported data for the relationship between *FCGR3A-V158F* rs396991 polymorphism and response to trastuzumab among Egyptian patients with breast cancer. Our study revealed that the frequencies of heterozygous genotypes (F/V), and homozygous genotypes (V/V) were statistically significantly higher in trastuzumab responders when compared to non-responders (F/V: 63.6% vs. 58.3% and V/V: 29.6% vs. 8.4%, respectively). Furthermore, we found that V allele carriers consistently exhibited a statistically significant better response among responders when compared to non-responders (V allele: 61.4% vs. 37.5%). On the other hand, the frequencies of the wild genotype (F/F) and (F) allele were statistically significantly higher among trastuzumab non-responders compared to responders (F/F: 33.3% vs. 6.8% and F allele: 62.5% vs. 38.6%, respectively). Also, a statistically significant difference was observed between various response grades and *FCGR3A-V158F* genotypes.

This was in agreement with the findings of *Musolino et al.* who studied 54 Italians with HER2-positive metastatic breast cancer who received trastuzumab and taxane, and they reported an improved response rate and for those patients having *FCGR3A-158* mutant V/V genotype^25^. Also, *Tamura et al.* studied *FCGR3A-V158F* polymorphism among the Japanese population, revealing a significant association between *FCGR3A-158* V/V and response rate among both early and metastatic breast cancer patients^26^.

Similar results were reported by *Gavin et al.*. in their retrospective study enrolling 1251 American patients who received adjuvant chemotherapy plus trastuzumab. They came to the same conclusion of the significant association of the *FCGR3A-V158F* polymorphism, the homozygous (*FCGR3A-158* V/V) patients received more benefit than those with *FCGR3A-158* F/F genotypes^15^.

Moreover, a randomized trial study conducted by the National Surgical Adjuvant Breast and Bowel Project trial on American and Canadian populations revealed that across all trastuzumab-based treatments patients with at least the presence of one *FCGR3A-158 V* of either V/V or F/V had statistically significantly higher complete response rate than patients who were homozygous for the low-affinity allele *FCGR3A-158 F* (F/F)^27^. This further confirms our results, showing that all patients with a complete response had *FCGR3A-158 V* of either V/V or F/V.

On the other hand, other studies replicated among various ethnicities concluded that the presence of the *FCGR3A-158* V allele was not associated with a better response rate or an improvement in disease-free survival among breast cancer patients. A cohort study carried out by *Hurvitz et al.*. on 1189 patients treated with trastuzumab-based therapy, revealing that there was no correlation between disease-free survival and *FCGR3A* genotypes^28^. Also, *Kim et al.*. reported similar negative results on 57 Korean metastatic breast cancer patients treated with taxane plus trastuzumab chemotherapy^29^. Moreover, *Roca et al.* did not find any correlation in their prospective study on a group of 132 patients from the UK^30^.

In addition, *Norton et al.* performed a study on 1,325 patients treated with adjuvant trastuzumab in their early stages of breast cancer. They revealed the absence of a significant difference in the clinical response or the improvement of disease-free survival between patients who were homozygous for *FCGR3A-V158F* rs396991 (V/V) and patients who were wild type (F/F)^24^. The conflict and variability in the results seen in the different studies may arise due to various possible factors such as ethnicity, sample sizes, and different tumor grades included in these studies.

Furthermore, *Gavin et al.* explained that the significant benefit from trastuzumab was limited to the *FCGR3A-158* V allele, which may be attributed to the fact that the *FCGR3A-158* V allele was associated with binding to IgG1 immune complexes at low concentrations, hence a better response to treatment. Meanwhile, the *FCGR3A-158* F allele did not show the same biological response^15^. This was further attributed to antibody-mediated ADCC activity, which is higher in patients with *FCGR3A-158* V/V genotypes than those with F/F genotypes, probably due in part to the greater affinity of the Fc region being reported among *FCGR3A-158* V than those with *FCGR3A-158* F^22^. However, the mechanism underlying the association between *FCGR3A-V158F* polymorphism and clinical response is not clearly understood.

Our study revealed statistically significant differences between the *FCGR3A-158* genotypes regarding OS and PFS. This was in agreement with *Musolino and colleagues*, who reported a better PFS and improved response rates for those patients with *FCGR3A-158* V/V among 54 patients with HER2-positive metastatic breast cancer who received trastuzumab and taxane^25^. This was further confirmed by *Tamura et al. who* assessed the PFS one year after the last patient’s enrollment in the study and revealed a statistical difference in the PFS of the patients with *FCGR3A-158* V/V in comparison with those having 158 F/V or F/F^26^.

In contrast, *Kim et al.* did not establish any correlation between *FCGR3A* and PFS in 57 patients treated with taxane plus trastuzumab chemotherapy^29^. They suggested that any differences attributed to the tested SNPs do not result in significant detectable differences in clinical outcomes^28,29^.

The different outcomes in multiple studies may be influenced by different studied populations or in different chemotherapy regimens conducted by different levels of aggressiveness of the disease, and by different sample sizes, sampling bias, and methodologies^22^.

In conclusion, our study reported a significant association between the *FCGR3A-V158F* polymorphism and response to trastuzumab-based therapy in breast cancer patients. This finding might provide a useful platform for clinical health practice for predicting the response to trastuzumab-based therapy and selecting those patients most likely to benefit from the avoidance of cost and unnecessary toxicity. Further work still needed to be performed to pinpoint the use of this SNP as a prognostic biomarker for assessing the response and progression of risk in breast cancer patients receiving trastuzumab-based regimens. Further studies with a larger sample size are needed to clarify the association.

Some limitations in our study should be addressed. Firstly, the sample size was relatively small, which could need further large studies. Secondly, the presence of other associated polymorphisms of the *FCGR3A* gene could affect the comprehensiveness and preciseness of the relationship between *FCGR3A-V158F* polymorphisms and clinical outcomes in breast cancer patients on trastuzumab-based therapy. Extended follow-up is needed to reach definite conclusions regarding PFS and OS. Finally, limited research articles, to some extent, especially those evaluating early non-metastatic breast cancer, could affect the validity of the results regarding the same studied population and the same chemotherapy-based treatment.

## Data Availability

The datasets used and/or analysed during the current study available from the corresponding author on reasonable request. The datasets of the sequences generated and/or analysed during the current study are available in the NCBI repository under the following numbers [2873805, 2873791].
